# 2-Amino-*N*,3-dimethyl­benzamide

**DOI:** 10.1107/S1600536812048027

**Published:** 2012-11-28

**Authors:** Xiang-dong Mei, Yan-hui Liang, Ke-Bin Li

**Affiliations:** a State Key Laboratory for Biology of Plant Diseases and Insect Pests, Institute of Plant Protection, Chinese Academy of Agricultural Sciences, Beijing 100193, People’s Republic of China

## Abstract

In the title compound, C_9_H_12_N_2_O, the mean plane through the amide group and the benzene ring form a dihedral angle of 33.93 (7)°. An intra­molecular N—H⋯O hydrogen bond is present. In the crystal, mol­ecules are linked by N—H⋯N and N—H⋯O hydrogen bonds, forming double-stranded chains parallel to the *b* axis.

## Related literature

For background to substituted anthranilamides, see: Gnamm *et al.* (2012 ▶); Lahm *et al.* (2005 ▶); Norman *et al.* (1996 ▶); Roe *et al.* (1999 ▶). For the synthesis, see: Staiger & Wagner (1953 ▶); Coppola (1980 ▶); Witt & Bergman (2000 ▶).
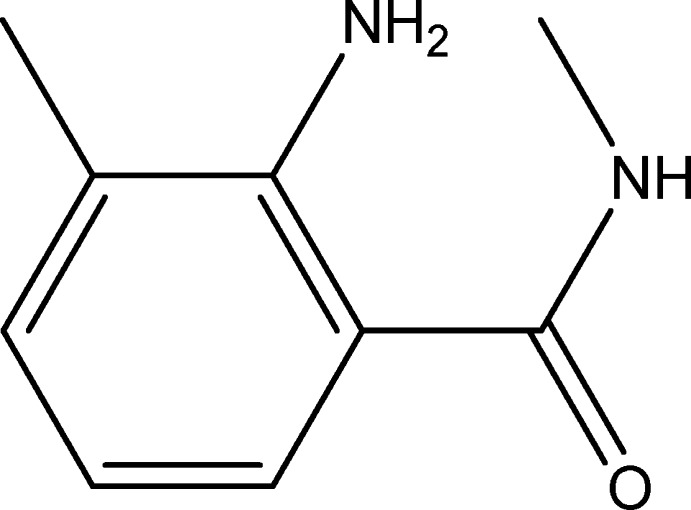


## Experimental

### 

#### Crystal data

C_9_H_12_N_2_O
*M* *_r_* = 164.21
Monoclinic, 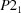

*a* = 9.833 (6) Å
*b* = 5.011 (3) Å
*c* = 9.841 (6) Å
β = 118.27 (1)°
*V* = 427.1 (4) Å^3^
*Z* = 2
Mo *K*α radiation
μ = 0.09 mm^−1^
*T* = 173 K
0.36 × 0.13 × 0.10 mm

#### Data collection

Rigaku MM007-HF CCD (Saturn 724+) diffractometer
Absorption correction: multi-scan (*CrystalClear*; Rigaku, 2007 ▶) *T*_min_ = 0.970, *T*_max_ = 0.992
3828 measured reflections
1939 independent reflections
1884 reflections with *I* > 2σ(*I*)
*R*_int_ = 0.036

#### Refinement

*R*[*F* ^2^ > 2σ(*F* ^2^)] = 0.042
*wR*(*F* ^2^) = 0.111
*S* = 1.05
1939 reflections
112 parameters
1 restraint
H-atom parameters constrained
Δρ_max_ = 0.30 e Å^−3^
Δρ_min_ = −0.22 e Å^−3^

### 

Data collection: *CrystalClear* (Rigaku, 2007 ▶); cell refinement: *CrystalClear*; data reduction: *CrystalClear*; program(s) used to solve structure: *SHELXS97* (Sheldrick, 2008 ▶); program(s) used to refine structure: *SHELXL97* (Sheldrick, 2008 ▶); molecular graphics: *OLEX2* (Dolomanov *et al.*, 2009 ▶); software used to prepare material for publication: *SHELXL97*.

## Supplementary Material

Crystal structure: contains datablock(s) I, global. DOI: 10.1107/S1600536812048027/rz5025sup1.cif

Structure factors: contains datablock(s) I. DOI: 10.1107/S1600536812048027/rz5025Isup2.hkl

Supplementary material file. DOI: 10.1107/S1600536812048027/rz5025Isup3.cml

Additional supplementary materials:  crystallographic information; 3D view; checkCIF report

## Figures and Tables

**Table 1:** Hydrogen-bond geometry (Å, °)

*D*—H⋯*A*	*D*—H	H⋯*A*	*D*⋯*A*	*D*—H⋯*A*
N1—H1*A*⋯O1	0.88	2.21	2.785 (2)	123
N1—H1*B*⋯N1^i^	0.88	2.44	3.240 (2)	151
N2—H2⋯O1^ii^	0.88	2.18	2.858 (2)	133

Symmetry codes: (i) 
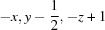
; (ii) 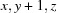.

**Fig. 1.**
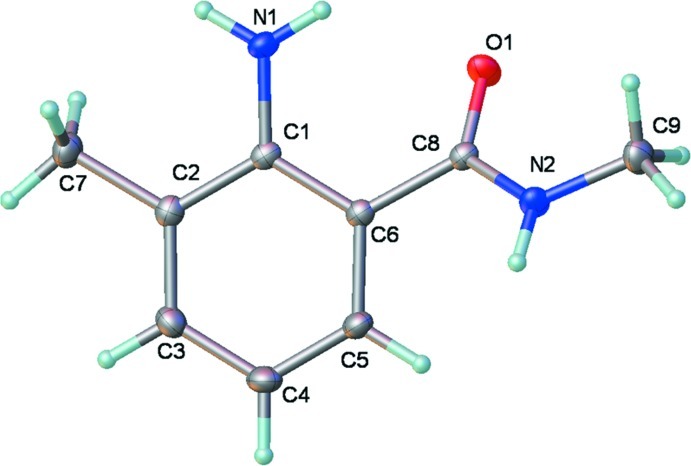
The molecular structure of the title compound showing 50% probability displacement ellipsoids and atom-numbering scheme.

## supplementary crystallographic information

### Comment

Anthranilamide-based derivatives exhibit interesting biological activities such as antibacterial, antifungal, antiviral and insecticidal effects (Gnamm *et al.*, 2012; Lahm *et al.*, 2005; Norman *et al.*, 1996; Roe *et al.*, 1999). We report here the crystal structure of the title compound 2-amino-*N*,3-dimethylbenzamide, an important organic intermediate in the synthesis of medicines, agricultural chemicals, and animal drugs.

In the title compound (Fig. 1), the least-square mean plane through the amide group (C8/C9/O1/N2) form a dihedral angle of 33.93 (7)° with the benzene ring. The molecular conformation is stabilized by an intramolecular N—H···O hydrogen bond (Table 1). In the crystal structure, intermolecular N—H···N and N—H···O hydrogen interactions link molecules into double chains running parallel to the *b* axis.

### Experimental

The title compound was prepared according to the literature method (Witt & Bergman, 2000) by stirring isatoic anhydride with aqueous methylamine. Isatoic anhydride was prepared by reaction of anthranilic acid with triphosgene in good yield (Coppola, 1980). The title compound (0.2 g) was dissolved in ethanol (50 ml) at room temperature. Colourless blocks were obtained through slow evaporation after two weeks.

### Refinement

The H atoms were placed at calculated positions, with C—H = 0.93–0.98 Å, and refined as riding with *U*_iso_(H) = 1.2–1.5*U*_eq_(C). 841 Friedel pairs were not merged.

### Crystal data

**Table:**

C_9_H_12_N_2_O	*F*(000) = 176
*M**_r_* = 164.21	*D*_x_ = 1.277 Mg m^−^^3^
Monoclinic, *P*2_1_	Mo *K*α radiation, λ = 0.71073 Å
Hall symbol: P 2yb	Cell parameters from 2240 reflections
*a* = 9.833 (6) Å	θ = 2.4–32.7°
*b* = 5.011 (3) Å	µ = 0.09 mm^−^^1^
*c* = 9.841 (6) Å	*T* = 173 K
β = 118.27 (1)°	Rod, colourless
*V* = 427.1 (4) Å^3^	0.36 × 0.13 × 0.10 mm
*Z* = 2	

### Data collection

**Table:**

Rigaku MM007-HF CCD (Saturn 724+) diffractometer	1939 independent reflections
Radiation source: rotating anode	1884 reflections with *I* > 2σ(*I*)
Confocal monochromator	*R*_int_ = 0.036
ω scans at fixed χ = 45°	θ_max_ = 27.5°, θ_min_ = 2.4°
Absorption correction: multi-scan (*CrystalClear*; Rigaku, 2007)	*h* = −12→12
*T*_min_ = 0.970, *T*_max_ = 0.992	*k* = −6→6
3828 measured reflections	*l* = −12→12

### Refinement

**Table:**

Refinement on *F*^2^	Primary atom site location: structure-invariant direct methods
Least-squares matrix: full	Secondary atom site location: difference Fourier map
*R*[*F*^2^ > 2σ(*F*^2^)] = 0.042	Hydrogen site location: inferred from neighbouring sites
*wR*(*F*^2^) = 0.111	H-atom parameters constrained
*S* = 1.05	*w* = 1/[σ^2^(*F*_o_^2^) + (0.0574*P*)^2^ + 0.1149*P*] where *P* = (*F*_o_^2^ + 2*F*_c_^2^)/3
1939 reflections	(Δ/σ)_max_ < 0.001
112 parameters	Δρ_max_ = 0.30 e Å^−^^3^
1 restraint	Δρ_min_ = −0.22 e Å^−^^3^

### Special details

**Table:**

Geometry. All e.s.d.'s (except the e.s.d. in the dihedral angle between two l.s. planes) are estimated using the full covariance matrix. The cell e.s.d.'s are taken into account individually in the estimation of e.s.d.'s in distances, angles and torsion angles; correlations between e.s.d.'s in cell parameters are only used when they are defined by crystal symmetry. An approximate (isotropic) treatment of cell e.s.d.'s is used for estimating e.s.d.'s involving l.s. planes.
Refinement. Refinement of *F*^2^ against ALL reflections. The weighted *R*-factor *wR* and goodness of fit *S* are based on *F*^2^, conventional *R*-factors *R* are based on *F*, with *F* set to zero for negative *F*^2^. The threshold expression of *F*^2^ > σ(*F*^2^) is used only for calculating *R*-factors(gt) *etc*. and is not relevant to the choice of reflections for refinement. *R*-factors based on *F*^2^ are statistically about twice as large as those based on *F*, and *R*- factors based on ALL data will be even larger.

### Fractional atomic coordinates and isotropic or equivalent isotropic displacement parameters (Å^2^)

**Table:**

	*x*	*y*	*z*	*U*_iso_*/*U*_eq_	
O1	0.18145 (16)	0.6816 (2)	0.25039 (14)	0.0310 (3)	
N1	0.08945 (16)	0.5876 (3)	0.47375 (16)	0.0267 (3)	
H1A	0.0480	0.5886	0.3725	0.032*	
H1B	0.0504	0.4853	0.5193	0.032*	
N2	0.22924 (17)	1.1192 (3)	0.24907 (16)	0.0276 (3)	
H2	0.2598	1.2682	0.3025	0.033*	
C1	0.21634 (17)	0.7472 (3)	0.56154 (18)	0.0215 (3)	
C2	0.27443 (18)	0.7589 (3)	0.72312 (19)	0.0245 (3)	
C3	0.3954 (2)	0.9304 (4)	0.80932 (19)	0.0300 (4)	
H3	0.4353	0.9365	0.9181	0.036*	
C4	0.4600 (2)	1.0941 (4)	0.7407 (2)	0.0325 (4)	
H4	0.5431	1.2102	0.8019	0.039*	
C5	0.40169 (18)	1.0856 (4)	0.58261 (19)	0.0266 (3)	
H5	0.4443	1.1990	0.5351	0.032*	
C6	0.28106 (17)	0.9128 (3)	0.49133 (17)	0.0211 (3)	
C7	0.2030 (2)	0.5880 (4)	0.7989 (2)	0.0330 (4)	
H7B	0.0947	0.6394	0.7603	0.050*	
H7C	0.2593	0.6139	0.9109	0.050*	
H7A	0.2085	0.3999	0.7748	0.050*	
C8	0.22609 (17)	0.8953 (3)	0.32129 (18)	0.0214 (3)	
C9	0.1841 (2)	1.1254 (4)	0.08586 (19)	0.0343 (4)	
H9A	0.0791	1.0551	0.0268	0.051*	
H9B	0.2555	1.0156	0.0663	0.051*	
H9C	0.1873	1.3098	0.0542	0.051*	

### Atomic displacement parameters (Å^2^)

**Table:**

	*U*^11^	*U*^22^	*U*^33^	*U*^12^	*U*^13^	*U*^23^
O1	0.0437 (7)	0.0195 (6)	0.0277 (6)	−0.0021 (5)	0.0152 (5)	−0.0023 (4)
N1	0.0268 (7)	0.0240 (6)	0.0291 (7)	−0.0067 (6)	0.0130 (5)	−0.0007 (6)
N2	0.0377 (8)	0.0188 (7)	0.0282 (7)	−0.0033 (6)	0.0173 (6)	−0.0010 (6)
C1	0.0194 (7)	0.0172 (7)	0.0290 (8)	0.0029 (6)	0.0124 (6)	0.0013 (6)
C2	0.0247 (8)	0.0230 (8)	0.0278 (8)	0.0043 (7)	0.0140 (7)	0.0030 (6)
C3	0.0287 (8)	0.0356 (9)	0.0235 (7)	−0.0004 (7)	0.0106 (7)	−0.0026 (7)
C4	0.0271 (8)	0.0344 (9)	0.0328 (8)	−0.0085 (8)	0.0115 (7)	−0.0077 (8)
C5	0.0260 (7)	0.0256 (7)	0.0314 (8)	−0.0056 (7)	0.0162 (7)	−0.0032 (7)
C6	0.0208 (7)	0.0185 (7)	0.0255 (7)	0.0018 (6)	0.0123 (6)	−0.0004 (6)
C7	0.0387 (9)	0.0323 (9)	0.0313 (8)	−0.0008 (8)	0.0192 (7)	0.0052 (8)
C8	0.0207 (7)	0.0181 (7)	0.0272 (7)	0.0016 (6)	0.0127 (6)	0.0007 (6)
C9	0.0431 (10)	0.0314 (10)	0.0286 (8)	−0.0010 (8)	0.0171 (8)	0.0043 (7)

### Geometric parameters (Å, º)

**Table:**

O1—C8	1.239 (2)	C3—H3	0.9500
N1—C1	1.386 (2)	C4—C5	1.381 (2)
N1—H1A	0.8800	C4—H4	0.9500
N1—H1B	0.8800	C5—C6	1.398 (2)
N2—C8	1.337 (2)	C5—H5	0.9500
N2—C9	1.450 (2)	C6—C8	1.498 (2)
N2—H2	0.8800	C7—H7B	0.9800
C1—C6	1.410 (2)	C7—H7C	0.9800
C1—C2	1.413 (2)	C7—H7A	0.9800
C2—C3	1.384 (2)	C9—H9A	0.9800
C2—C7	1.510 (2)	C9—H9B	0.9800
C3—C4	1.392 (3)	C9—H9C	0.9800
			
C1—N1—H1A	120.0	C6—C5—H5	119.5
C1—N1—H1B	120.0	C5—C6—C1	119.49 (14)
H1A—N1—H1B	120.0	C5—C6—C8	120.10 (14)
C8—N2—C9	122.35 (15)	C1—C6—C8	120.36 (14)
C8—N2—H2	118.8	C2—C7—H7B	109.5
C9—N2—H2	118.8	C2—C7—H7C	109.5
N1—C1—C6	121.07 (15)	H7B—C7—H7C	109.5
N1—C1—C2	119.33 (14)	C2—C7—H7A	109.5
C6—C1—C2	119.43 (14)	H7B—C7—H7A	109.5
C3—C2—C1	119.16 (14)	H7C—C7—H7A	109.5
C3—C2—C7	121.00 (16)	O1—C8—N2	121.14 (14)
C1—C2—C7	119.84 (15)	O1—C8—C6	121.59 (14)
C2—C3—C4	121.71 (15)	N2—C8—C6	117.26 (14)
C2—C3—H3	119.1	N2—C9—H9A	109.5
C4—C3—H3	119.1	N2—C9—H9B	109.5
C5—C4—C3	119.12 (16)	H9A—C9—H9B	109.5
C5—C4—H4	120.4	N2—C9—H9C	109.5
C3—C4—H4	120.4	H9A—C9—H9C	109.5
C4—C5—C6	121.09 (15)	H9B—C9—H9C	109.5
C4—C5—H5	119.5		
			
N1—C1—C2—C3	−176.23 (16)	N1—C1—C6—C5	175.30 (15)
C6—C1—C2—C3	−0.9 (2)	C2—C1—C6—C5	0.1 (2)
N1—C1—C2—C7	3.0 (2)	N1—C1—C6—C8	−7.4 (2)
C6—C1—C2—C7	178.30 (16)	C2—C1—C6—C8	177.33 (13)
C1—C2—C3—C4	0.8 (3)	C9—N2—C8—O1	−1.2 (3)
C7—C2—C3—C4	−178.38 (18)	C9—N2—C8—C6	177.79 (15)
C2—C3—C4—C5	0.1 (3)	C5—C6—C8—O1	144.60 (17)
C3—C4—C5—C6	−1.0 (3)	C1—C6—C8—O1	−32.7 (2)
C4—C5—C6—C1	0.9 (3)	C5—C6—C8—N2	−34.4 (2)
C4—C5—C6—C8	−176.35 (16)	C1—C6—C8—N2	148.33 (15)

### Hydrogen-bond geometry (Å, º)

**Table:**

*D*—H···*A*	*D*—H	H···*A*	*D*···*A*	*D*—H···*A*
N1—H1*A*···O1	0.88	2.21	2.785 (2)	123
N1—H1*B*···N1^i^	0.88	2.44	3.240 (2)	151
N2—H2···O1^ii^	0.88	2.18	2.858 (2)	133

Symmetry codes: (i) −*x*, *y*−1/2, −*z*+1; (ii) *x*, *y*+1, *z*.
